# Towards a biomarker panel for the assessment of AKI in children receiving intensive care

**DOI:** 10.1007/s00467-015-3089-3

**Published:** 2015-04-15

**Authors:** James McCaffrey, Beatrice Coupes, Chris Chaloner, Nicholas J. A. Webb, Rachael Barber, Rachel Lennon

**Affiliations:** 1 Department of Paediatric Nephrology, Royal Manchester Children’s Hospital, Central Manchester University Hospitals NHS Foundation Trust (CMFT), Manchester Academic Health Science Centre, Manchester, UK; 2 Department of Renal Medicine, CMFT, Manchester Academic Health Science Centre, Manchester, UK; 3 Paediatric Intensive Care Unit, CMFT, Manchester Academic Health Science Centre, Manchester, UK; 4 Department of Biochemistry, CMFT, Manchester Academic Health Science Centre, Manchester, UK; 5 Institute of Human Development, Faculty of Medical and Human Sciences, University of Manchester, Manchester, UK

**Keywords:** Acute kidney injury, Paediatric intensive care unit, Biomarkers, Cystatin C, Neutrophil gelatinase-associated lipocalin, Kidney injury molecule-1

## Abstract

**Background:**

Critically ill children and neonates are at high risk of developing acute kidney injury (AKI). AKI is associated with short- and long-term renal impairment and increased mortality. Current methods of diagnosing AKI rely on measurements of serum creatinine, which is a late and insensitive marker. Few studies to date have assessed AKI biomarkers in a heterogeneous patient cohort.

**Methods:**

We conducted a prospective feasibility study in a paediatric intensive care setting over a 6-month period to describe the relationship between AKI (defined according to pRIFLE criteria) and new AKI biomarkers.

**Results:**

In total, 49 patients between the ages of 16 days and 15 years were recruited for measurement of plasma cystatin C (Cys-C) and neutrophil gelatinase-associated lipocalin (pNGAL) concentrations, as well as for urinary kidney injury molecule-1 (KIM-1) and urinary NGAL (uNGAL) concentrations. Almost one-half (49 %) of the patient cohort experienced an AKI episode, and Cys-C and pNGAL were the strongest candidates for the detection of AKI. Our data suggest that the widely used estimated baseline creatinine clearance value of 120 mL/min/1.73 m^2^ underestimates actual baseline function in patients admitted to paediatric intensive care units.

**Conclusions:**

This investigation demonstrates the feasibility of new AKI biomarker testing in a mixed patient cohort and provides novel biomarker profiling for further evaluation.

**Electronic supplementary material:**

The online version of this article (doi:10.1007/s00467-015-3089-3) contains supplementary material, which is available to authorized users.

## Introduction

Acute kidney injury (AKI) is a serious condition characterized by the sudden onset of renal dysfunction, leading to impaired control of acid–base, electrolyte and fluid balance. AKI is a common problem in children admitted to the paediatric intensive care unit (PICU). The incidence of AKI following admission to PICU varies from 4.5 to 82 %, with higher incidence in children requiring invasive mechanical ventilation and/or vasoactive medications [1–4]. The most comprehensive longitudinal paediatric follow-up study currently available was limited by follow-up rates of less than 50 %, but it reported overall 3- to 5-year post-AKI patient survival of only 57 %, with 9 % of surviving children developing end-stage renal disease by 5 years [1].

Despite the clinical significance of AKI, current diagnosis and treatment strategies remain suboptimal. Diagnosis of AKI relies on serum measurements of muscle-derived creatinine, which is efficiently excreted by the healthy kidney. However, baseline serum creatinine (SCr) excretion shows wide variation associated with age, sex, muscle mass and other inter-individual variability [2]. In addition, SCr levels only rise above baseline when 25–50 % of renal function has been lost [3], and there may be a temporal dissociation between rises in SCr and true renal filtration [5]. The ability to diagnose AKI earlier in the disease course would allow a more timely instigation of interventions to prevent secondary damage (such as dose-adjustment or elimination/avoidance of nephrotoxic medicines, avoidance of intravenous contrast agents and appropriate fluid management) and, where applicable, earlier initiation of renal replacement therapy. Additionally, current evaluation of novel therapeutic strategies is hampered by treatment initiation only commencing after significant renal damage has occurred [6].

AKI in paediatric patients is classified using the criteria of the paediatric RIFLE classification system for AKI [pRIFLE (Risk, Injury, Failure, Loss, End-stage kidney disease)] [4]. The pRIFLE classification/staging system stratifies AKI based on urine output and changes in estimated creatinine clearance (eCCl) rather than the absolute SCr value. In addition to the development of AKI classification systems, there has been a long-standing search for novel biomarkers to enable earlier detection of AKI. A number of candidate biomarkers were identified following the discovery and identification of renal genes that are rapidly upregulated following a period of renal ischaemia [7, 8]. These include human neutrophil gelatinase-associated lipocalin (NGAL) [9–12], cystatin-C (Cys-C) [13, 14] and kidney injury molecule-1 (KIM-1) [12, 15].

AKI biomarker studies in the paediatric population have to date focused on selected groups of patients, such as children undergoing cardiopulmonary bypass procedures [13, 16] or following the onset of haemolytic uraemic syndrome [10], and a number of studies have exclusively focussed on neonates [11, 14, 15]. Of those studies that have examined more general admissions, data are only provided for urinary biomarkers [12, 17, 18] or exclusively in the neonatal period [14]. A recent study involving 214 children admitted to PICUs with sepsis showed that a combination of three plasma AKI biomarkers and clinical risk stratification strongly predicts the development of severe renal injury in children with sepsis [19].

Here we present data from a feasibility study, which is the first to examine both urinary and plasma biomarkers at multiple time points in a general PICU population. The aims of our study were: (1) to identify biomarkers that differentiate patients with and without AKI in a heterogeneous paediatric cohort; (2) to examine whether biomarkers provide an earlier warning of AKI than pRIFLE criteria; (3) to investigate whether a combination of biomarkers improves the identification of AKI.

## Methods

### Study design and participants

The study group was a prospective cohort of patients admitted either electively or as an emergency to the PICU at the Royal Manchester Children’s Hospital (RMCH) between December 2011 and June 2012. Subjects younger than 16 years of age admitted to the PICU who required the insertion of a central venous or peripheral arterial line and urinary catheter as part of their routine clinical care were eligible for recruitment. Patients whose parents or guardians did not consent or those with stage 4 or 5 chronic kidney disease or who were dialysis dependent prior to their PICU admission were excluded; no patients who had undergone previous renal transplantation were recruited. The acceptability of using deferred consent in this population was explored.

### Ethical approval and consent

The study received ethical approval from the National Research Ethics Committee (ref: 11/NW/0553) and was sponsored by Central Manchester University Hospitals NHS Foundation Trust (ref: R01708). Written consent was obtained for every participant from their parents or guardians. Special approval was granted for consent to be obtained retrospectively up to 48 h after admission, at which point sample collection had already commenced.

### Measurement of biomarkers

Blood and urine samples were collected twice daily from the time of PICU admission for a maximum period of 7 days. Samples were stored at 4 °C post-collection for a maximum of 16 h before transfer to the laboratory for processing. Stability of our proposed biomarkers has previously been demonstrated over this time period [20, 21]. Laboratory processing of blood samples involved centrifugation at 2000 rpm for 10 min to separate the plasma, which was aliquoted and stored at −80 °C. Laboratory processing of urine samples involved centrifugation at 2000 rpm for 5 min, followed by aliquoting of the samples and storage at −80 °C. All samples were batched for analysis of biomarkers.

NGAL and KIM-1 were measured by colourimetric immunoassay using commercially available reagents following the manufacturer’s guidelines. Plasma NGAL (pNGAL) and urine NGAL (uNGAL) were assayed using the R&D Systems (Oxford, UK) duoset DY1757. R&D immunoassay reagents (DLCN20) have been reported to demonstrate satisfactory performance for all parameters tested in a rigorous evaluation of NGAL measurement [22]. Urine KIM-1 (uKIM-1) was assayed using the R&D Systems duoset DY1750. A detailed evaluation of this assay for uKIM-1 demonstrated reliability in a standard clinical research setting [21]. Assay plates were read on a SPECTRAmax 340PC (Molecular Devices, Sunnyvale, CA). Sample concentrations in all assays were calculated from a 4-parameter standard curve (SOFTmax PRO v4 software; Molecular Devices). In both assays an intra-assay coefficient of variation (CV) of <10 % was confirmed in our hands, and an internal standard included on each assay plate confirmed an inter-assay CV of <20 % throughout the duration of the study. Biomarker concentrations in urine were corrected for Cr concentration [measured by a modified version of the 555 creatinine assay kit (Sigma Aldrich, St. Louis, MO), using the Jaffe reaction) and expressed as units/mg Cr.

Plasma Cys-C concentration was measured using a particle-enhanced immunoturbidimetric assay (Tina-quant; Roche Diagnostics, Mannheim, Germany) on a Roche Cobas 6000 modular instrument (Roche Diagnostics) using the manufacturer’s assay configuration and settings. Calibrators (CfAS) and control materials were used as supplied with the Roche kits. Cys-C concentration was measured in heparinized plasma samples after separation from the cells by centrifugation and transfer to a secondary analytical cup. The between-batch coefficients of variation established from daily repeated measures of control material were 1.37 % at 1.27 mg/L and 1.21 % at 4.49 mg/L (*n* = 26 each). The manufacturer’s reported measuring range is 0.4–8.0 mg/L.

### Definition of AKI

Patients were classified using the pRIFLE criteria for AKI which assess a combination of changes in eCCl from baseline eCCl and absolute urine output values [4]. The pRIFLE Risk ®) stage is defined by a 25 % decrease in eCCl ± urine output of <0.5 mL/kg/h for 8 h; the pRIFLE Injury (I) stage is defined by a 50 % decrease in eCCl ± urine output of <0.5 mL/kg/h for 16 h; the pRIFLE Failure (F) stage is defined by a 75 % decrease in eCCl or an eCCl of <35 mL/min/1.73 m^2^ ± urine output of <0.3 mL/kg/h for 24 hr or anuria for 12 h.

**Fig. 1:**
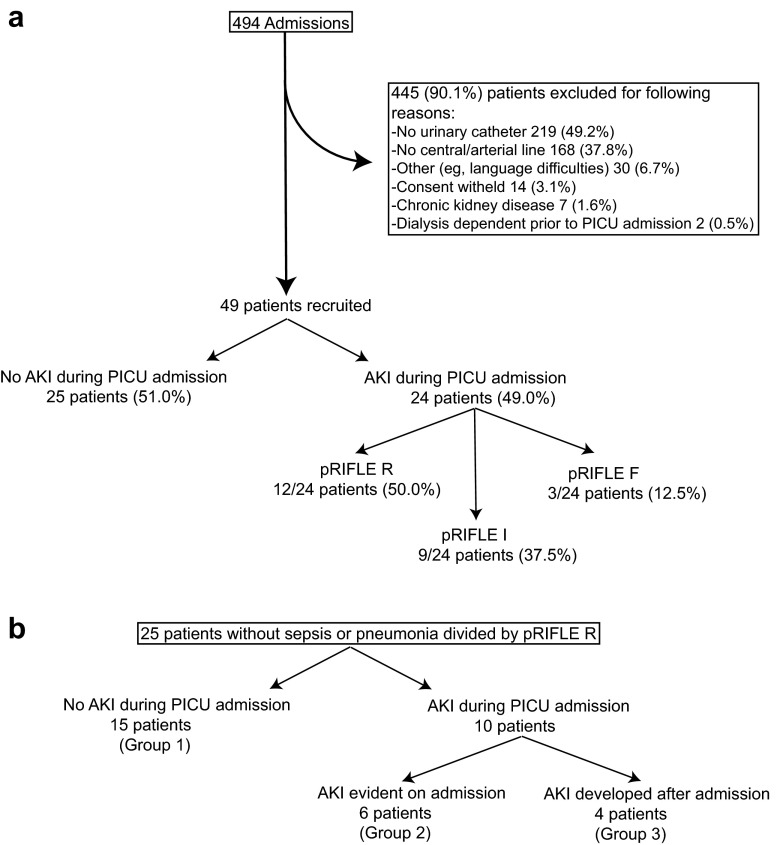
Patient recruitment and acute kidney injury (*AKI*) classification. **a** Total patient cohort, **b** patients admitted with diagnosis other than sepsis or pneumonia. *pRIFLE* Pediatric RIFLE (Risk, Injury, Failure, Loss, End-stage kidney disease) classification system for AKI, *PICU* paediatric intensive care unit

eCCl was calculated in the same way as the estimated glomerular filtration rate in children, i.e. using the Schwartz formula calculation with a *k* coefficient of 36.5, in keeping with our local practice [23]. Baseline kidney function was defined as the lowest known SCr value in the 3 months prior to hospital admission. Patients with no known prior SCr values available were assigned a baseline eCCl of 120 mL/min/1.73 m^2^ [12, 19]. As shown in Fig. 1 (overview of patient recruitment), patients were assigned to groups on the basis of the most severe pRIFLE stratum they reached during PICU admission (pRIFLEmax) on either SCr (pRIFLE_SCr_) or urine output (pRIFLE_UOP_) criteria. For subsequent analyses, biomarker values were allocated to pRIFLE strata in a time-specific manner. For example, biomarker values in the ‘pRIFLE R’ group included any biomarker reading at any time, for any patient, for which that patient at that time was reaching the inclusion criteria for pRIFLE R. Each individual biomarker reading was only included in a single pRIFLE stratum, an approach which avoided pre-insult and post-recovery biomarker values confounding the results.

### Statistical analysis

The correlations between biomarker levels and SCr were analysed by calculating a Spearman non-parametric correlation coefficient (*r*). SCr levels were first normalized for patient height using the Schwartz equation (effectively calculating an eCCl). The correlation coefficient ranges from −1 to +1. A correlation coefficient of 0 signifies that there is no correlation between biomarker levels, while a value of −1 or +1 signifies perfect correlation [24]. Biomarker levels were also compared between different pRIFLE strata. Continuous variables were assessed by non-parametric testing (Mann–Whitney *U* test for two groups and the Kruskal–Wallis test followed by Dunn’s multiple comparison test for three groups or more). We determined receiver operator characteristic (ROC) curves and calculated the areas under the curves (AUCs) for the four biomarkers analysed in this study. ROC curves were compared using the method of Hanley and McNeil [25]. An AUC of 0.5 is no better than expected by chance, whereas a value of 1.0 signifies a perfect biomarker. All analyses were performed using GraphPad Prism version 5.04 for Windows (GraphPad Software, San Diego CA).

## Results

### Cohort characteristics and incidence of AKI

Over the study period, there were 494 admissions to the PICU (Fig. 1a), from which a total of 49 patients were recruited into the study. The most common cause for study ineligibility was the absence of a urinary catheter (219/445, 49.2 %) or central venous or peripheral arterial line (168/445, 37.8 %); the presence of a urinary catheter and central venous or peripheral arterial line were necessary for sample collection. This inclusion requirement led to recruitment of a cohort of particularly unwell PICU patients. Summary characteristics are presented in Table 1. Of the 49 patients, 26 (53 %) were female, and 25 (58.1 %) were White British; the median age of the entire patient cohort was 3 (range 0.04–15) years. The most common reason for admission was pneumonia (12/49, 24.5 %) and sepsis (12/49, 24.5 %). Ventilation was required by 43/49 (88 %) patients. The mortality rate was 5/49 (10.2 %). Of the 49 patients, four (8.2 %) were admitted electively, none of whom experienced an AKI episode, and 24 (49.0 %) experienced an AKI episode during PICU admission. Twelve patients reached pRIFLEmax R, nine patients reached pRIFLEmax I and three patients reached pRIFLEmax F (Fig. 1a).

**Table 1:** Baseline characteristics and clinical details of patient cohort

Patient baseline and clinical characteristics	Number (%)	Median	Iinterquartile range	Range
Age (years)		3	1–7	0.04–15.00
Sex				
Female	26 (53)			
Male	23 (47)			
Height (cm)		95.0	74.0–120.0	44.0–173.0
Weight (kg)		12.9	8.0–28.5	1.9–70.0
Ethnicity	43 available			
White British	25 (58.1)			
Other White	6 (14.0)			
Pakistani	9 (20.9)			
Other Asian background	3 (7.0)			
Diagnosis at admission				
Pneumonia	12 (24.5)			
SIRS/sepsis/shock	12 (24.5)			
Cardiac arrest	5 (10.2)			
Elective surgical	4 (8.2)			
Emergency Surgical	4 (8.2)			
Trauma	3 (6.1)			
Seizures	2 (4.1)			
Other	7 (14.3)			
Pre-existing illness				
None	16 (32.6)			
Neuromuscular	2 (4.1)			
Genetic	7 ( 14.3)			
Seizures	3 ( 6.1)			
Other	21 (42.8)			
Ventilated (days)	43 (87.7)	5	3–9	2–14
Death	5 (10.2)			
CVVH	1 (2)			
Inotrope score	44			
Highest		0	0–9	0–61
Lowest		0	0–0	0–13
PELOD score	44			
Highest		13	11–21	0–41
Lowest		1	0–11	0–20
Blood products				
Whole blood	18 (37.5)			
Fresh frozen plasma	11 (22.9)			
Cryoprecipitate	1 (2.0)			
Platelets	3 (6.3)			
Urine output (mL/kg/h)				
Highest		7.1	5.4–11.1	0.9–15.3
Lowest		0.5	0.4–0.7	0.0–1.4
Nephrotoxic medication	33 (67.3)			
Aminoglycosides	9 (18.4)			
Vancomycin	1 (2.0)			
Teicoplanin	4 (8.2)			
Furosemide	24 (49.0)			
Nonsteroidal anti-inflammatory drugs	8 (16.3)			

CVVH, Continuous veno-venous hemofiltration, PELOD, Paediatric Logistic Organ Dysfunction

### Baseline creatinine clearance

**Fig. 2:**
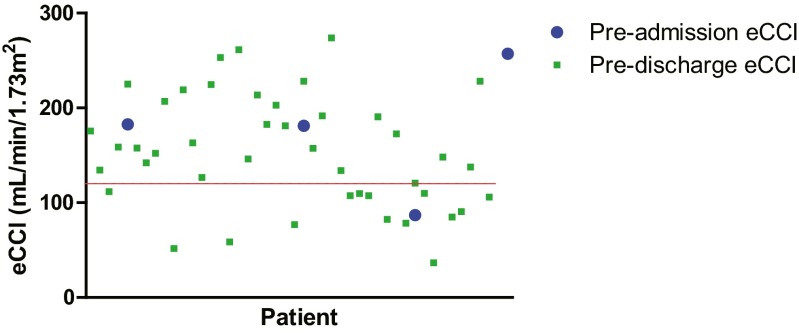
Comparison of pre-admission and pre-discharge estimated creatinine clearance (*eCCl*) values for patients enrolled in the study. *Red line* Presumed eCCl value of 120 mL/min/1.73 m^2^ used in the paediatric RIFLE classification for acute kidney injury

In previous AKI studies in children, a presumed baseline eCCl (calculated using the Schwartz formula) of 120 mL/min/1.73 m^2^ has been used in the absence of pre-admission SCr readings [12, 19]. In our study, we examined whether an eCCl of 120 mL/min/1.73 m^2^ was representative of baseline renal function in our patient cohort. We obtained pre-admission and pre-discharge SCr values for study subjects to calculate eCCls. Pre-admission SCr values were recorded if available in the 3-month period prior to hospital admission. Pre-discharge SCr values were defined as the final SCr recording prior to hospital discharge, although this may have been affected by muscle loss during the PICU admission. We excluded patients who died prior to PICU discharge. Only 4/49 (8.2 %) of our patient cohort had pre-admission SCr values, and Fig. 2 shows that the majority of pre-admission (75.0 %) and pre-discharge (68.2 %) eCCl values were above the presumed baseline value of 120 mL/min/1.73 m^2^. These data suggest that the widely used presumed baseline eCCl value of 120 mL/min/1.73 m^2^ may underestimate baseline renal function in our patient cohort.

### Biomarkers responsive to changes in eCCl

**Fig. 3:**
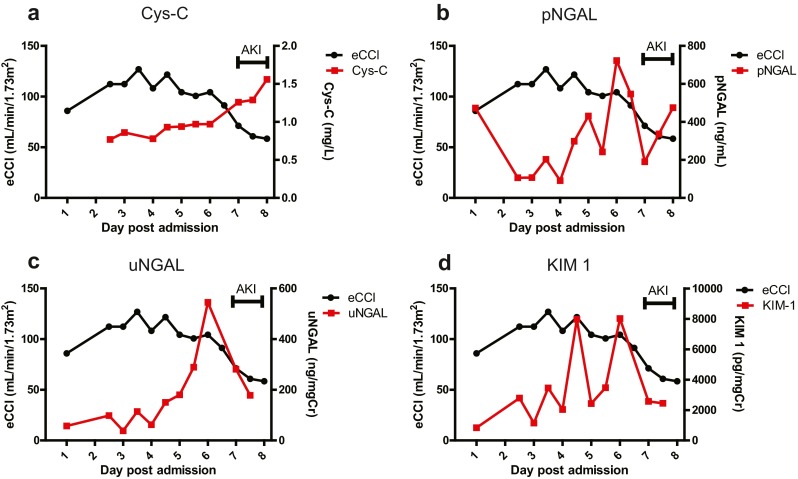
Biomarker profiles plots for a patient with acute kidney injury (AKI) according to the most severe pRIFLE stratum reached by the patient during PICU admission (pRFILEmax). This patient reached the pRIFLEmax Injury (I) stratum at day 7 after admission to the PICU. *Black filled circles/lines* eCCl (mL/min/1.73 m^2^), *red filled squares/lines* biomarker levels. **a** cystatin-C (*Cys-C*; mg/L), **b** plasma neutrophil gelatinase-associated lipocalin (*pNGAL*; ng/mL), **c** urinary NGAL (*uNGAL*; ng/mg Cr), **d** kidney injury molecule-1 (*KIM-1*; pg/mg Cr). Note that the period of AKI is given on the top right of each plot. As eCCl declines over time, the level of each of biomarker increases

Biomarker profile plots were created for each patient to aid visualization of data, and an example plot from a patient with AKI (pRIFLEmax I) is shown in Fig. 3. This profile demonstrates an increase in Cys-C coincident with falling eCCl and peaks in pNGAL, uNGAL and KIM-1 prior to the decrease in eCCl. A representative set of biomarker plots from a patient not experiencing AKI is provided in Electronic Supplementary Material Fig. 1.

To determine whether eCCl values from all patients correlated with coincident biomarker values, we calculated a Spearman non-parametric correlation coefficient (*r*) for each biomarker. A significant correlation was observed for Cys-C (*r* = −0.77, *p* < 0.0001), a weak correlation was observed for pNGAL (*r* = −0.14, *p* = 0.043) and uNGAL (*r* = −0.13, *p* = 0.045), and there was no significant correlation with coincident eCCl and the urinary biomarker KIM-1 (*r* = −0.076, *p* = 0.26).

### Biomarkers elevated in AKI

**Fig. 4:**
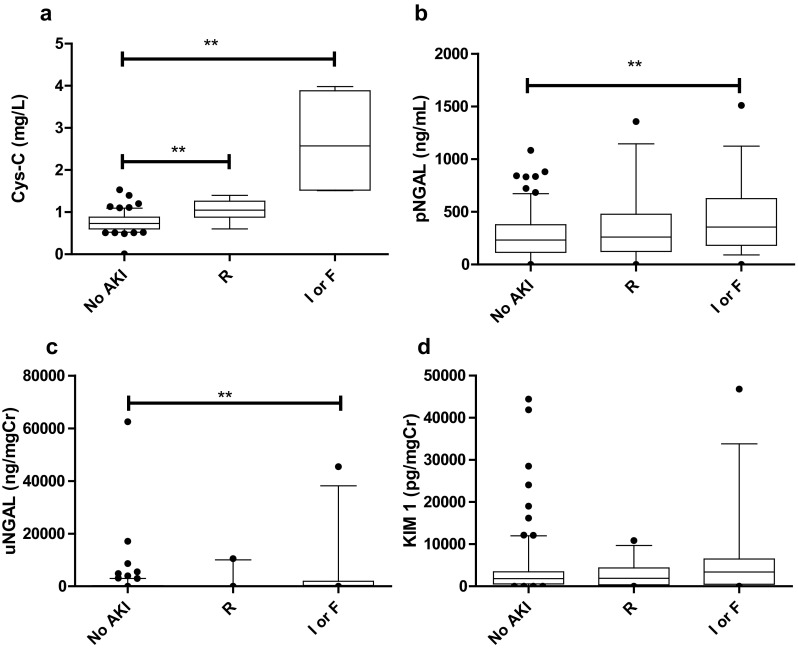
Box and whisker graph showing biomarker levels according to pRFILE stratum. **a** Cys-C levels were significantly higher during periods of pRIFLE Risk (*R*) compared to no acute kidney injury (AKI) (*No AKI*), and during periods of pRIFLE Injury or Failure (*I or F*) compared to pRIFLE R (*p* < 0.05). **b**, **c** pNGAL (**b**; *p* = 0.027) and uNGAL (**c**; *p* = 0.0079) levels were significantly higher in periods of pRIFLE I or F compared to no AKI. **d** No significant difference in KIM-1 levels were observed (*p* = 0.2108). In all plots, the box extends from the 25th to 75th data percentiles; *line in the middle of box* is plotted at the median, *box plot whiskers* denote 5th and 95th data percentiles

We investigated whether biomarker levels changed according to AKI severity. For the three groups we considered (no AKI, pRIFLE R, pRIFLE I/F), we found that pNGAL (*p* = 0.027) and uNGAL (*p* = 0.0079) levels were significantly higher in periods of pRIFLE I/F (Fig. 4) and that Cys-C levels were significantly higher in periods of both pRIFLE R and pRIFLE I/F (*p* < 0.0001). This result suggests that the biomarker levels of Cys-C, pNGAL and uNGAL may aid the diagnosis of AKI.

### Biomarker sensitivity and specificity

**Fig. 5:**
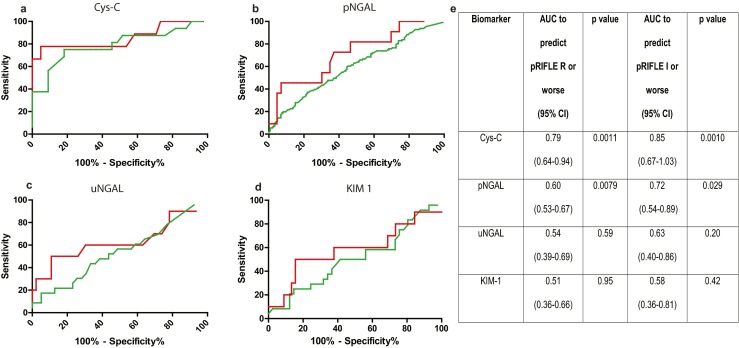
Receiver operating characteristic (ROC) curve analysis was performed to evaluate the ability of Cys-C (**a**), pNGAL (**b**), uNGAL (**c**) and KIM-1 (**d**) to diagnose acute kidney injury (AKI). *Green lines* ROC curves showing biomarker sensitivity and specificity if an AKI definition of pRIFLE R or worse is used, *red lines* ROC curves illustrating biomarker performance if an AKI definition of pRFILE I or worse is used. **e** Area under the curve (*AUC*) values. *CI* Confidence interval

To identify which of the biomarkers provided the highest sensitivity and specificity, we performed an ROC analysis using both ‘pRIFLE R or worse’, and ‘pRIFLE I or worse’ to define AKI. All biomarkers displayed superior predictive power for ‘pRIFLE I or worse’ compared to ‘pRIFLE R or worse’. The best performing biomarkers were Cys-C and pNGAL (Fig. 5). We also considered the variation of Cys-C with age. It has been reported that infants under the age of 18 months have a higher mean Cys-C value than older children [26]. To determine if this pattern held true in our patient cohort, we compared median Cys-C values of patients not experiencing AKI under 18 months old to those older than 18 months and found no significant difference (≤18 months 0.75 mg/L, ≥18 months 0.78 mg/L; *p* = 0.66). We additionally examined whether a correlation between fluid status and biomarker levels existed. No significant correlation was observed for Cys-C, pNGAL or uNGAL, although a weak correlation was observed for KIM-1 (ESM Fig. 2).

### The utility of pNGAL in AKI associated with sepsis

**Fig. 6:**
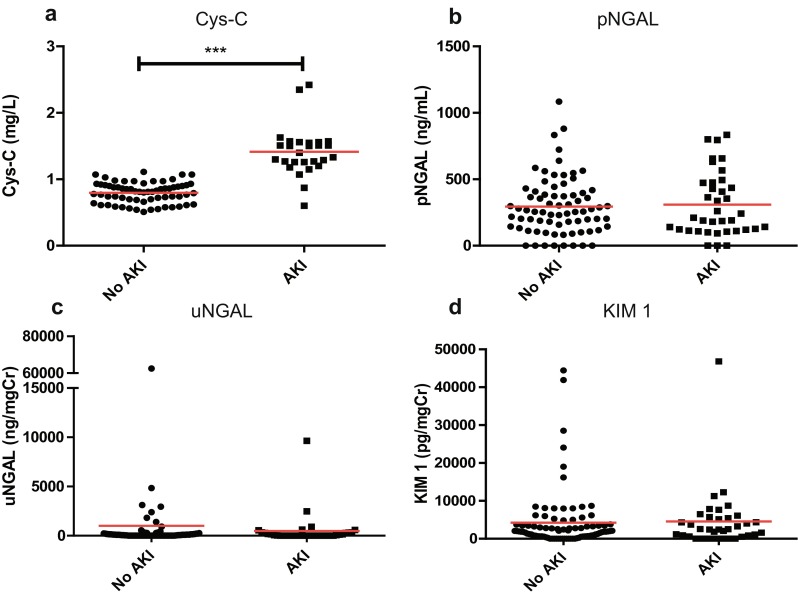
Biomarker levels in periods of no acute kidney injury (AKI) compared to periods of AKI in patients admitted to PICU with sepsis or pneumonia. Cys-C (**a**), pNGAL (**b**), uNGAL (**c**) and KIM-1 (**d**) in periods of AKI (defined by pRIFLE R or worse) in this patient group. There was no significant difference in pNGAL levels during AKI (*p* value 0.97). *Red lines* Mean biomarker values. *** *p* < 0.0001

It has previously been reported that Cys-C and uNGAL levels are not altered by sepsis, whereas pNGAL levels rise in sepsis and cannot reliably discriminate AKI from no AKI in the septic state [27]. As 24/49 (49.0 %) of our patients were admitted to the PICU with either sepsis or pneumonia, we examined whether pNGAL was a valid AKI biomarker in sepsis in our patient cohort. For patients admitted to PICU with either sepsis or pneumonia, we compared biomarker levels during periods of no AKI to periods of AKI using an AKI definition of pRIFLE R or worse (Fig. 6). Whereas Cys-C retained its ability to discriminate no AKI from AKI (*p* < 0.0001), there was no significant difference in pNGAL levels between no AKI and AKI periods in patients admitted with sepsis or pneumonia (*p* =0.97). We also performed an ROC analysis using median biomarker values for patients admitted to the PICU with either sepsis or pneumonia, using pRIFLE R as a cut-off between no AKI and AKI. Again, Cys-C retained its utility as an AKI biomarker in this patient group (AUC 0.86, *p* = 0.002), while pNGAL performed poorly (AUC 0.54, *p* = 0.71). Thus, our data support the previous data indicating that pNGAL is not a reliable biomarker for AKI associated with sepsis.

### Identification of biomarker cut-off levels

By examining biomarker levels in periods of no AKI and AKI (Fig. 4) and the ROC analysis (Fig. 5), we concluded that Cys-C and pNGAL were the best performing biomarkers to diagnose AKI in our study group. We proceeded to identify biomarker cut-off values, including Cys-C and pNGAL for patients without sepsis or pneumonia, and Cys-C alone for patients with sepsis or pneumonia. Proposed biomarker cut-off levels were set according to the sensitivities and specificities provided by the ROC analyses. From these analyses we identified a Cys-C value of >0.91 mg/L (75 % sensitivity, 82 % specificity) and a pNGAL value of >258 ng/mL (88 % sensitivity, 62 % specificity) as cut-off levels for AKI.

### A biomarker panel improves specificity for AKI

To test the hypothesis that biomarker specificity may be improved by introducing a requirement for abnormal Cys-C and pNGAL values to be recorded at the same time, patients without sepsis or pneumonia were split into three groups (Fig. 1b). pNGAL was not considered here as our results had already shown that it is not a reliable AKI marker in patients with sepsis or pneumonia. Group 1 contained patients not experiencing an AKI episode during PICU admission, Group 2 contained patients experiencing AKI on PICU admission (to test the hypothesis in patients with established AKI) and Group 3 consisted of patients with no AKI evident on PICU admission but who subsequently experienced an AKI episode during PICU admission (to test the hypothesis in patients prior to AKI onset). For this analysis, patients with pRIFLE R or worse were considered to have AKI. We found that the combination of Cys-C and pNGAL improved the specificity of the test [92.9 %, compared with the use of either Cys-C or pNGAL alone (85.7 % and 57.1 % respectively)] (Table 2). For patients in Group 2 (established AKI), this improved specificity came without a loss of sensitivity (compared to either test in isolation). However, contemporaneous combination of the two biomarkers in Group 3 (prior to AKI) reduced sensitivity to only 20 %. One possible explanation is that Cys-C and pNGAL peak at different times prior to an episode of AKI. We therefore proceeded to examine the temporal profiling of these biomarkers.

**Table 2:** Assessment of the change in sensitivity and specificity of acute kidney injury biomarkers using contemporaneous measurements in patients admitted with a diagnosis other than sepsis or pneumonia compared to single biomarkers in isolation

Biomarker	Sensitivity (%)	Specificity (%)
Cys-C alone (in Group 2)	100	85.7
pNGAL alone (in Group 2)	100	57.1
C + P (in Group 2)	100	92.9
Cys-C alone (in Group 3)	60	85.7
pNGAL alone (in Group 3)	80	57.1
C + P (in Group 3)	20	92.9

C + P, Contemporaneous abnormal cystatin C (Cys-C) and plasma human neutrophil gelatinase-associated lipocalin (pNGAL)

For definition of groups, see Fig. 1b and section A biomarker panel improves specificity for acute kidney injury (AKI)

### Temporal profiling of plasma biomarkers

We investigated the time course of Cys-C and pNGAL levels in the period prior to AKI onset for the four patients in Group 3 who were admitted to PICU without sepsis or pneumonia. Using the cut-off values of >0.91 mg/L for Cys-C and >258 ng/mL for pNGAL, an abnormal pNGAL value was recorded in three of these patients prior to AKI onset, while none of the patients had an abnormal Cys-C reading prior to AKI onset. Additionally, the highest recorded pNGAL level in a given patient’s biomarker profile occurred prior to AKI onset in all four patients, compared to two of the four patients for Cys-C. In this limited data set, pNGAL levels tended to peak earlier than Cys-C levels and were often declining by the time the Cys-C levels began to rise.

### Temporal profiling of urinary biomarkers

The urinary biomarkers uNGAL and KIM-1 displayed a poor ability to diagnose AKI in the general patient group (Fig. 5). However, in an exploratory analysis, we examined the possibility that these biomarkers may have utility in predicting future AKI using data from all patients developing AKI after admission to PICU. We identified urinary biomarker values from six patients (pRIFLE R or worse) in the 24 h prior to an AKI episode and compared these to median values in the 24 h post-AKI. Five of these six patients had lower uNGAL levels in the 24 h after AKI diagnosis, while five displayed higher KIM-1 levels in the 24 h after AKI diagnosis. The possibility that uNGAL may peak before the diagnosis of AKI using pRIFLE criteria and thus act as an early marker of AKI could be explored in a future, appropriately powered study.

### Patients with AKI and normal eCCl

Three patients in the study reached pRIFLE AKI criteria on the basis of low urine output alone (i.e. had normal eCCl). Biomarker profile plots are provided in ESM Figs. 3–5. No pNGAL plot is provided in ESM Fig. 5 since this patient was admitted with sepsis. The remaining two patients both had pNGAL readings which exceeded our proposed pNGAL cut-off value of 258 ng/mL prior to AKI diagnosis. All three patients recorded their highest uNGAL level prior to AKI diagnosis. Again, this result suggests that pNGAL and uNGAL may be earlier and more sensitive markers of AKI than SCr.

## Discussion

In this investigation, we tested a panel of plasma and urinary biomarkers to diagnose AKI in a general PICU setting. We have demonstrated that recruitment and sample collection is feasible using a retrospective consent model in a mixed patient cohort. We provide evidence that Cys-C and pNGAL are useful biomarkers for established AKI without sepsis and that Cys-C remains a valid biomarker for established AKI with sepsis. Contemporaneous readings of Cys-C and pNGAL improve the specificity without affecting the sensitivity in diagnosing established AKI without sepsis compared to either biomarker alone, and we provide preliminary data that pNGAL and uNGAL may be useful early markers of AKI.

In our patient cohort, Cys-C levels did not provide earlier warning of an AKI episode than SCr. However, Cys-C measurements may be of practical benefit in diagnosing AKI when no baseline SCr level is available for a patient. Cys-C levels are not dependent on muscle mass, and it is therefore possible to avoid one of the major pitfalls of an SCr-based AKI diagnosis [28]. Our ROC analysis suggests that a Cys-C cut-off value of 0.91 mg/L may be useful to identify AKI.

In our patient group, both Cys-C and pNGAL performed well individually for diagnosing established AKI (without sepsis), and we suggest a cut-off value of 258 ng/mL for pNGAL. However, the specificity of the biomarkers was improved by using combined contemporaneous measurements. Indeed, a biomarker panel may ultimately improve our current ability to diagnose AKI based on eCCl.

Several studies to date have used an eCCl of 120 mL/min/1.73 m^2^ as a presumed baseline value for children without a documented pre-admission eCCl. We found that pre-admission eCCl values were only available in 8.2 % of our patient cohort; this may be more of an issue in paediatric practice compared to adult practice, since children are more likely to be healthy prior to their hospital admissions. As a consequence, a majority of children in our study were presumed to have a baseline eCCl of 120 mL/min/1.73 m^2^. Previous studies have also noted a low proportion (24 %) of paediatric emergency admissions having a pre-recorded eCCl value [18] and also a high mean baseline eCCl of 154 mL/min/1.73 m^2^ for a mixed cohort of children admitted to an ICU [4]. Our data suggest that an eCCl value of 120 mL/min/1.73 m^2^ underestimates the actual baseline renal function for a majority of children and may lead to underdiagnosis of AKI.

In summary, we suggest that Cys-C has immediate utility for confirming a diagnosis of AKI in children admitted to intensive care. pNGAL and uNGAL may peak before the onset of AKI and therefore have the potential to predict episodes of AKI, although there needs to be caution with the use of pNGAL in sepsis. We propose that future AKI biomarker studies in a general PICU setting should differentiate patients admitted with sepsis from those admitted for other indications. Such a study would be the next step towards developing a biomarker panel, which in combination with risk stratification, could identify those children who need to be protected from secondary renal injury during their inpatient admission.

## Electronic supplementary material

Below is the link to the electronic supplementary material.

Supplementary Figure 1 (GIF 46 kb)

High resolution image (EPS 950 kb)

Supplementary Figure 2 (GIF 32 kb)

High resolution image (EPS 1217 kb)

Supplementary Figure 3 (GIF 44 kb)

High resolution image (EPS 947 kb)

Supplementary Figure 4 (GIF 48 kb)

High resolution image (EPS 968 kb)

Supplementary Figure 5 (GIF 32 kb)

High resolution image (EPS 876 kb)
