# Supplementary figures and images for: Towards a biomarker panel for the assessment of AKI in children receiving intensive care
Source: Pediatr Nephrol. 2015 Apr 15;30(10):1861–71. doi: 10.1007/s00467-015-3089-3 (PMC4549390; doi:10.1007/s00467-015-3089-3)

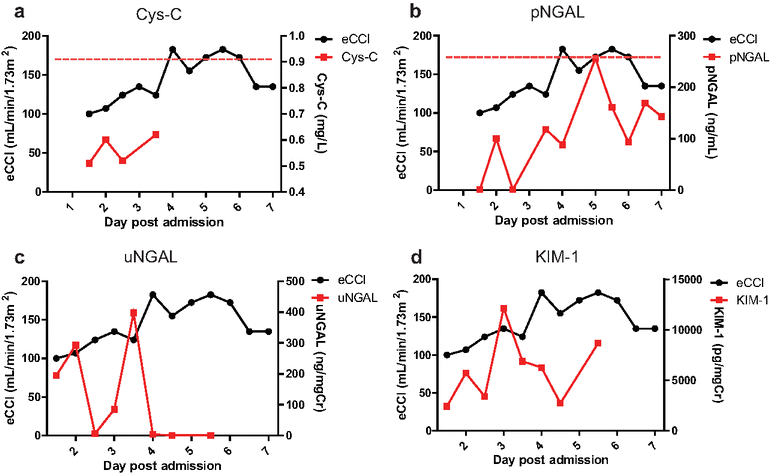

Supplement: Supplementary file 1 — (GIF 46 kb) [file 467_2015_3089_Fig7_ESM.gif]

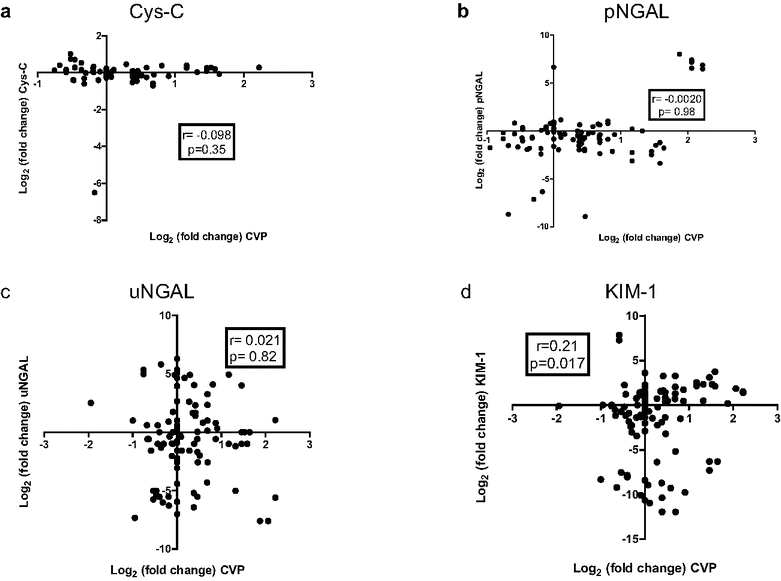

Supplement: Supplementary file 3 — (GIF 32 kb) [file 467_2015_3089_Fig8_ESM.gif]

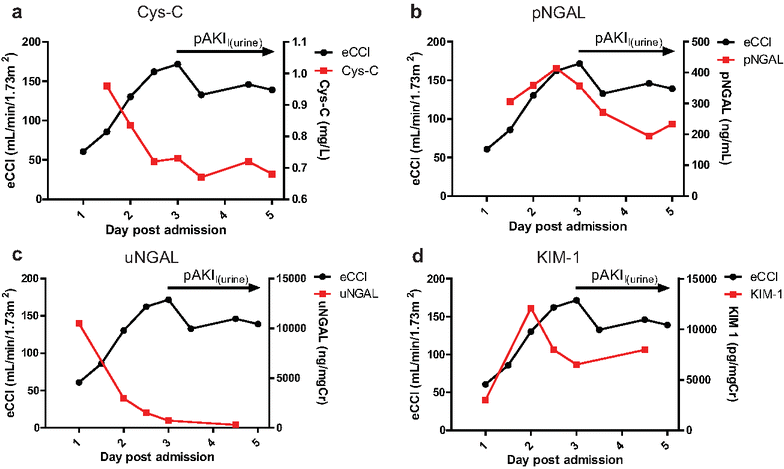

Supplement: Supplementary file 5 — (GIF 44 kb) [file 467_2015_3089_Fig9_ESM.gif]

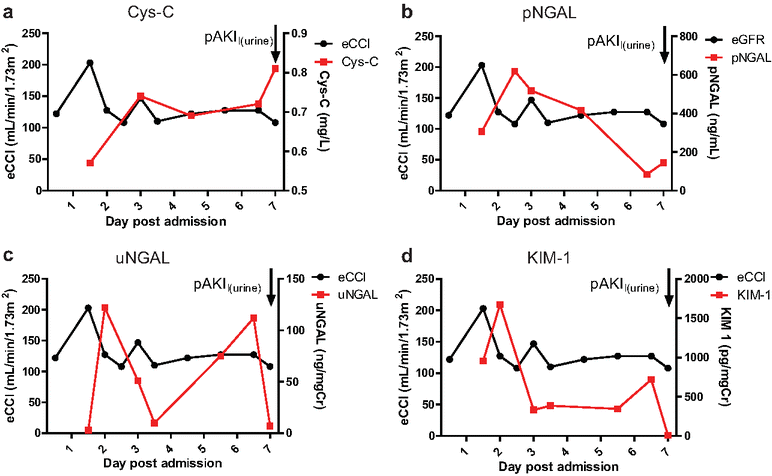

Supplement: Supplementary file 7 — (GIF 48 kb) [file 467_2015_3089_Fig10_ESM.gif]

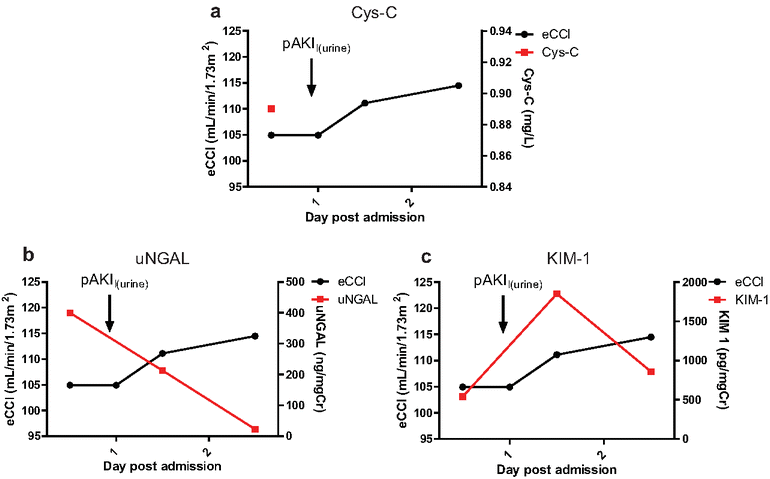

Supplement: Supplementary file 9 — (GIF 32 kb) [file 467_2015_3089_Fig11_ESM.gif]
